# Application of the prenatal BACs-on-Beads™ assay for rapid prenatal detection of sex chromosome mosaicism

**DOI:** 10.1007/s00438-022-01931-0

**Published:** 2022-07-28

**Authors:** Min Zhang, LingJi Chen, Meihuan Chen, Yan Wang, Bin Liang, Na Lin, Xiaoqing Wu, Linshuo Wang, Liangpu Xu, Hailong Huang

**Affiliations:** Medical Genetic Diagnosis and Therapy Center of Fujian Maternity and Child Health Hospital College of Clinical Medicine for Obstetrics and Gynecology and Pediatrics, Fujian Medical University, Fujian Provincial Key Laboratory of Prenatal Diagnosis and Birth Defect, Fuzhou City, 350001 Fujian Province China

**Keywords:** Prenatal diagnosis, Sex chromosome mosaicism, BACs-on-Beads array, Karyotyping, SNP-array, CNV-seq

## Abstract

The prenatal BACs-on-Beads™ (BoBs) assay was introduced for rapid detection of abnormalities of chromosomes 13, 18, 21, X, and Y and specific nine significant microdeletion syndromes. The ability of prenatal BoBs to detect mosaicism ranged from 20 to 40%. However, there have been no prenatal studies of sex chromosome mosaicism in prenatal BoBs. Therefore, the present study was performed with an aim to uncover the detection level of sex chromosome mosaicism that application of prenatal BoBs assay, and then to assess the sensitivity of prenatal BoBs assay, thereby improving the prenatal diagnostic accuracy. A total of 31 samples of amniotic fluid (AF) and umbilical cord blood (UCB) for prenatal diagnosis were collected, and the results were confirmed through karyotyping, single nucleotide polymorphism microarray (SNP-array) and copy number variation sequencing (CNV-seq). 23 cases of sex chromosome mosaicism were prompted abnormal by prenatal BoBs, the minimum detection level of mosaicism was about 6% as detected by karyotype. The overall sensitivity of prenatal BoBs in the detection of sex chromosome mosaicism was 74.2% (23/31). This study evaluated the effectiveness of prenatal BoBs for detecting sex chromosome mosaicism in prenatal diagnosis, and the results will provide valuable information for genetic counseling.

## Introduction

Chromosomal abnormality is one of the leading causes of fetal malformation and early pregnancy loss (Hyde and Schust 2015). Chromosomal mosaicism is observed in 0.3–1.5% of fetuses who were prenatally diagnosed with chromosomal abnormalities, and sex chromosome anomalies exist in 48% of chromosomal mosaicism cases (Hsu et al. 1996). As such, sex chromosome abnormalities are present in ~ 0.5% of live births (Zheng et al. 2019), and the prevalence of 45,X mosaicism in northeast China is 0.36% (Liu et al. 2013). Prenatal diagnoses of sex chromosome mosaicism present a particular counseling challenge, as the broad clinical spectrum ranges from a classic appearance with many physical differences from unaffected individuals to having minimal or no apparent observable features (Kamel et al. 2015; Tokita and Sybert 2016; Tuke et al. 2019; Weidler et al 2019). Therefore, to properly manage the clinical mosaicism, the following problems need to be solved: how to correctly evaluate the reliability of the mosaicism results, and how to do further analysis for confirmation of the mosaicism.

Recently, molecular cytogenetic methodologies have improved our methods of detecting fetal chromosome anomalies. At present, prenatal BACs-on-Beads™ (BoBs™) is used in clinics for the rapid detection of aneuploidies of chromosomes 13, 18, 21, X, and Y. It was also designed to detect gains and losses of DNA in chromosomal regions associated with nine microdeletion syndromes (Vialard et al. 2011). At present, few studies have been published to validate the reliability of these assays with regard to mosaicism (Cheng et al. 2013; Donaghue et al. 2005; Lund et al. 2019; Schouten et al. 2002; Shen et al. 2018). The ability of prenatal BoBs to detect mosaicism at various targeted regions ranges from 20 to 40% (Cheng et al. 2013). However, no studies have yet been done on prenatal diagnoses of sex chromosome mosaicism using prenatal BoBs. As such, our aim was to complete a comparative study on the ability of prenatal BoBs to detect and diagnose sex chromosome mosaicism, and provide genetic consulting for the population at risk.

## Materials and methods

### Materials

A total of 31 fetal DNA samples identified as sex chromosome mosaicism were selected from the samples previously analyzed by karyotype methods that had DNA samples available from July2016 to October2020, at Fujian Maternity and Child Health Hospital, Fuzhou, Fujian Province, People’s Republic of China (PRC). This study was approved by the Clinical Ethics Committee (2020KY195) of the Fujian Maternity and Child Health Hospital. Of these 31 samples, 21 (67.7%) were from amniotic fluid (AF) samples and 10 (32.3%) were from umbilical cord blood (UCB) samples. The proportion of mosaicism range from 4.8% to 94%. The clinical indications for prenatal diagnosis were performed in a high-risk pregnancy condition, such as advanced maternal age, increased maternal serum screening risk, fetal ultrasound anomalies, increased sex chromosome aneuploidy of non-invasive prenatal testing risk and previous fetus/child with abnormality. All of the 31 samples were simultaneously tested by the prenatal BoBs, and some were verified by single nucleotide polymorphism array (SNP-array) and copy number variation sequencing (CNV-seq) simultaneously, especially with small marker chromosomes.

## Methods

### Karyotype analysis

Amniotic fluid or fetal blood was examined by G-banding karyotyping with two independent cultures. Samples were disaggregated mechanically and enzymatically with collagenase II (Worthington, Freehold, NJ, USA). The chromosomes of samples were examined using the standard G-banding method with Wright's stain (Sigma-Aldrich; St Louis, MO, USA). Five metaphase cells were carefully examined by an experienced technician to detect structural chromosomal abnormalities, and at least 20 metaphase cells were examined for numerical chromosomal abnormalities. Metaphases were analyzed and karyograms prepared using CytoVision, a computer-assisted karyotyping system (Leica Biosystems, Newcastle, UK). The karyotyping were described according to the International System for Human Cytogenetics Nomenclature (2020).

### DNA extraction

Genomic DNA was extracted from cord blood and amniocentesis was performed using the QIAGEN DNA Mini Kit (Qiagen, Hilden, Germany) following manufacture's instructions. The extracted DNA was quantified by a NanoDrop 2000 Spectrophotometer (Thermo Fisher Scientific, MA, USA) to ensure DNA concentration was > 50 ng/μl, and the 260/280 nm optical density ratio was 1.8–2.0.

### Prenatal BoBs assay

Genomic DNA was obtained from AF or UCB using the QIAamp DNA Blood Mini kit (QIAGEN, Hilden, Germany). The prenatal BoBs assay was performed using a prenatal chromosome aneuploidy and microdeletion detection test kit (Perkin Elmer, Waltham, MA, USA), according to the manufacturer’s instructions. Genomic DNA from the specimens and reference DNA were first marked with biotin using an enzymatic method. A purification test kit was then used to purify the marked genomic DNA. After purification, the mixture of marked genomic DNA and BoBs™ has subjected to hybridization overnight. The microbeads were washed after hybridization and were then incubated with the reporter molecule (streptavidin–phycoerythrin). Using Luminex 200 (Austin, TX, USA) flow cytometry instrument to measure the fluorescence of DNA, and BoBsoft™ analytical software (Perkin Elmer) was used for data analysis. The ratio of specimen fluorescence to reference fluorescence was calculated. According to the manufacturer, a ratio greater than 1.0 indicated that the chromosome fragments were repeated and a ratio of less than 1.0 indicated a deletion. Five probe sites were set in each X and Y chromosome regions (Table 1).

**Table 1:** Targeted regions of X and Y chromosome probes detected by prenatal BoBs

Target area	Target number	Chromosome location	Fragment start position (Kb)	Fragment end position (Kb)	Linear central position (Mb)
Chromosome X	1	Xp22.31	6958	7137	7,05
	2	Xp22.2	10,699	10,763	10,73
	3	Xp21.1	37,368	37,555	37,46
	4	Xq13.2	73,303	73,450	73,38
	5	Xq27.3	146,757	146,960	146,86
Chromosome Y	1	Yp11.2	8461	8636	8,55
	2	Yq11.221	15,036	15,174	15,10
	3	Yq11.222	19,299	19,472	19,39
	4	Yq11.223	25,313	25,468	25,39
	5	Yq11.23	25,489	25,646	25,57

### SNP-array

Genomic DNA was obtained from amniocytes or fetal cord blood using the QIAamp DNA Blood Mini kit (QIAGEN, Hilden, Germany). Chromosomal microarray analysis was performed with the use of the Affymetrix CytoScan 750 k (Affymetrix, Santa Clara, CA, USA), which includes 550,000 nonpolymorphic probes for determination of copy-number changes and 200,000 single nucleotide polymorphism probes for allelic confirmation of copy number changes and detection of loss of heterozygosity. Genomic DNA was digested with Nsp enzyme and filled in with primer for PCR amplification. After purification, a fragmentation reaction was performed to form a fragment of about 25 bases, which is labeled with biotin and hybridized with the probe. After washing, streptavidin–phycoerythrin staining was used to detect fluorescence signals by scanning. Array analyses were performed using the Chromosome Analysis Suite software (ChAS), version 3.3 (Affymetrix, Santa Clara, CA, USA). The quality control (QC) parameters were applied according to the manufacturer´s recommendations. Samples with MAPD ≤ 0.25, SNP quality control (SNPQC) ≥ 15 (or ≥ 12 when all other parameters met the requirements), and waviness standard deviation (waviness SD) ≤ 0.12 were also included in the study. Autosomal CNVs that had a minimum coverage of 50 probes and a minimum size of 200 kb for gains and 100 kb for losses were considered for the analysis of pathogenicity. We just reported CNV of 400 kb, if cases that are highly suspicious of a clinically relevant condition, CNVs were reported even if < 400 Kb. The genomic imbalances were annotated based on the GRCh37/hg19 Genome Build (Feb 2009). CNVs analysis was performed using the DGV (http://dgv.tcag.ca/dgv/app/home), DECIPHER (https://decipher.sanger.ac.uk/), ClinVar (https://www.ncbi.nlm.nih.gov/clinvar/), ClinGen Dosage Sensitivity Map (https://dosage.clinicalgenome.org), OMIM (https://omim.org/) for analysis of genes associated with diseases. CNVs were finally classified as (1) Pathogenic (2) Likely Pathogenic (3) Variants of uncertain significance (VOUS) (4) benign (5) likely benign, following the 2019 American College of Medical Genetics(ACMG) guidelines.

### CNV-seq

In brief, 2 ml of amniotic fluid or umbilical cord blood was collected and centrifuged for 10 min at room temperature. The TIANamp Genomic DNA Kit (Tiangen, Beijing, China) was then used according to the user’s instruction to extract fetal gDNA, followed by library construction, pooling, sequencing, and quality control (NextSeq 550AR; Annoroad, Beijing, China). For each sample, around 7 million single end reads were sequenced and each read is about 35 bp long. After trimming and cleaning, a Burrows–Wheeler Aligner was used to map the processed reads to the human reference genome of UCSC genome annotation database (hg19 assembly) and a 100-kb window was applied to dived chromosomes into small sections from p to q arms. In each window, uniquely mapped reads were counted followed by GC-content correction and locally weighted scatter plot smoothing. An in-house reference database was then used to determine if the testing samples contains chromosome anomalies and the pathogenicity of CNVs was evaluated based on the Online Mendelian Inheritance in Man (OMIM), Database of Genomic Variation and Phenotype in Humans Using Ensembl Resources (DECIPHER), ClinGen, 1000 genomes, Database of Genomic Variants and American College of Medical Genetics guideline.

## Results

### Overall data

**Fig. 1:**
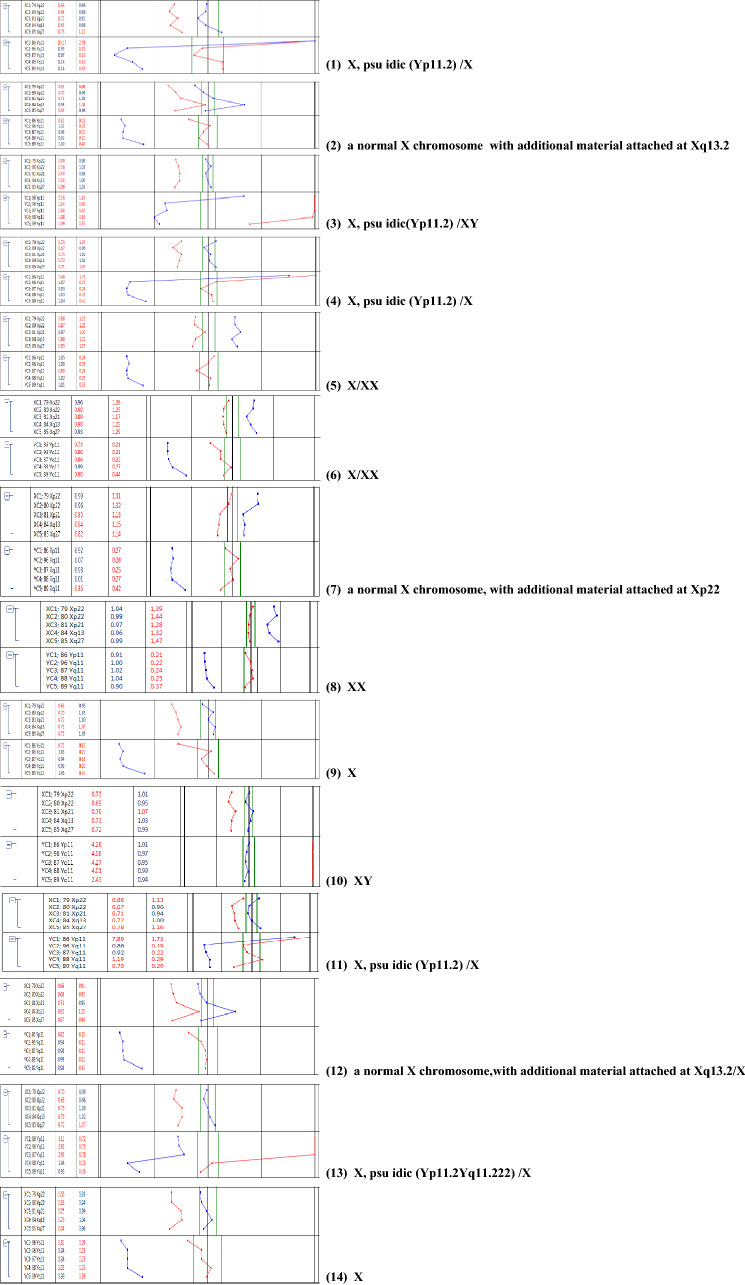

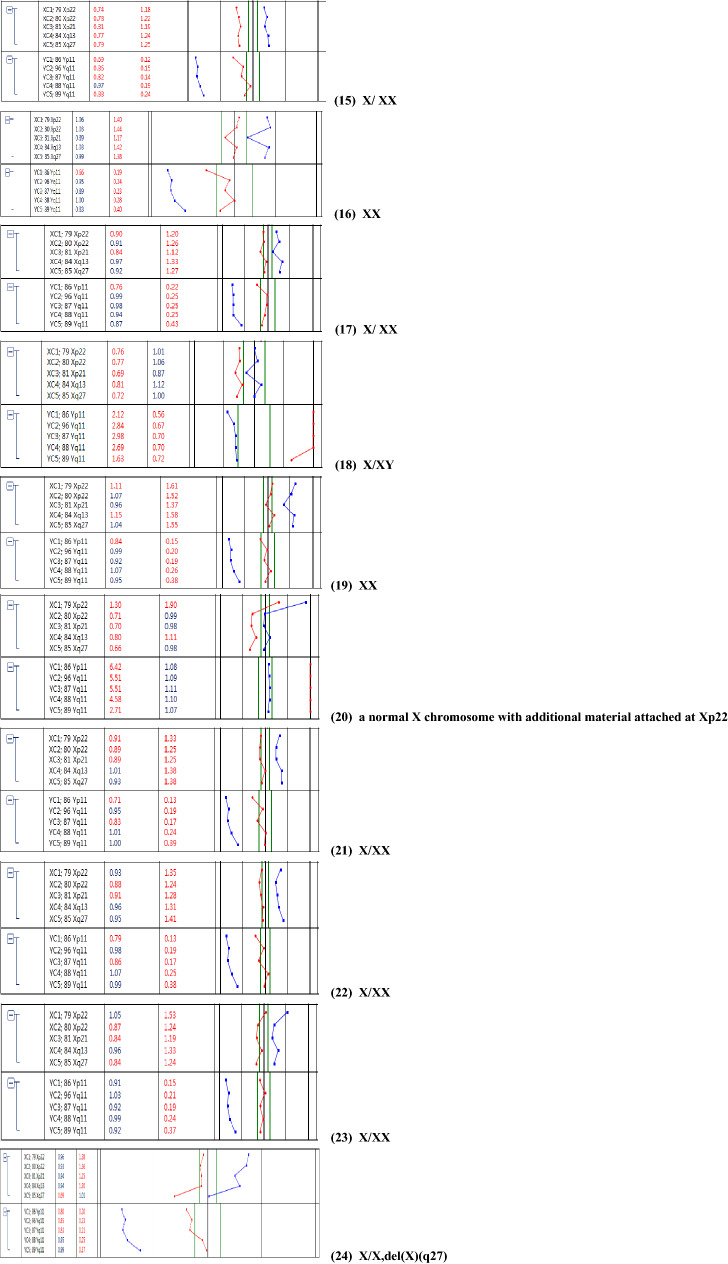

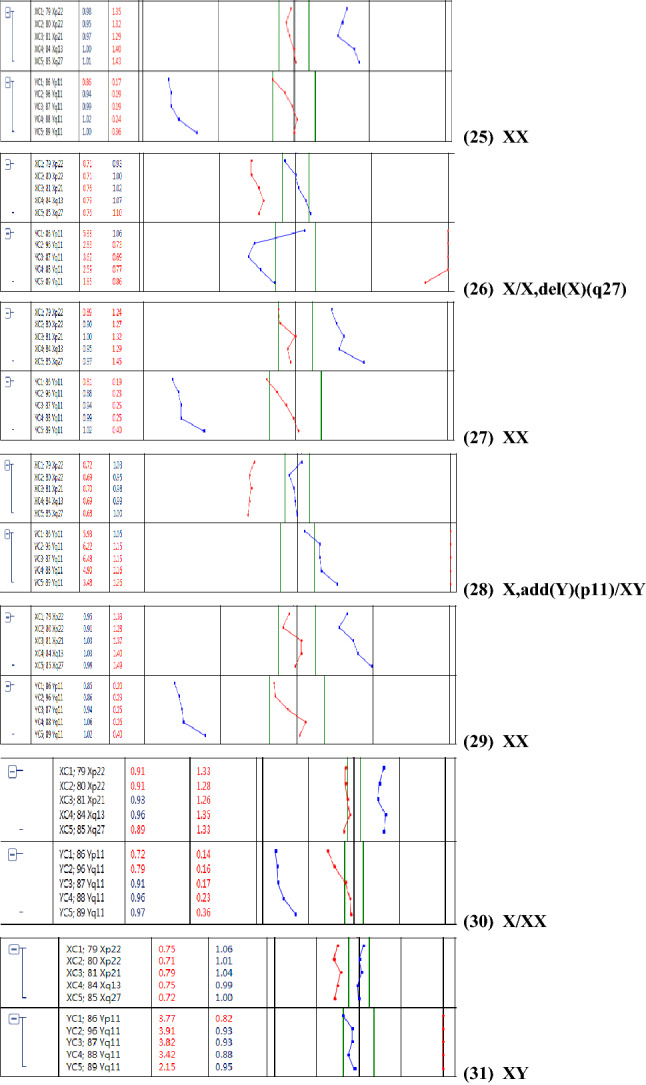
Results of sex chromosome by prenatal BoBs assay. The Results tab shows for each sample a numeric and graphic representation of probe and group ratios against female (F) and male (M) references. Red color in a graph indicates the ratio against female (Sample/F), while blue indicates the ratio against male (Sample/M), numerical ratios exceeding user defined thresholds are highlighted with red color

A total of 31 samples from pregnant women, consisting of 21 AF samples and 10 UCB samples, were evaluated for sex chromosome mosaicism. 19 of 31 samples (61.2%) had abnormal mosaicism, 4 (12.9%) had structurally abnormal mosaicism, and 8 (25.8%) had mosaicism of small supernumerary marker chromosomes (sSMC). There were 23 cases of sex chromosome abnormality diagnosed by prenatal BoBs and the sensitivity is approximate 74.2% (23/31). Notably, the detection of mosaicism by BoBs was as low as 6%. Among these 23 cases, sex chromosomes were abnormal and the results suggested mosaicism in 19 cases, and only suggested sex chromosomes abnormality but not mosaicism in 4 cases (cases 2, 9, 14, and 20). No obvious abnormalities were observed in the other 8 samples with mosaicism of sSMCs, with the rate of sex chromosome mosaicism ranging from 4.8% to 38.7% (Table 2; Fig. 1).

**Table 2:** Results of sex chromosome mosaicism detected by prenatal BoBs

Case	Sample type	Karyotype	Level of mosaiciam	BoBs results	SNP-array results	CNV-seq results	Pregnancy outcomes
1	UCB	46，X,+mar[86]/45,X[14]	86%/14%	X, idic (Y) (p11.2) /X mosaicism	The copy number of Yq11.221 q11.23 region is 0, refers to the fragment size of 10.7 Mb. The copy number of Yp11.31 q11.221 region is 4, refers to the fragment size of 15.3 Mb	The copy number of Yq11.221q11.23 region is 0, refers to the fragment size of 10.1 Mb. The copy number of Yp11.31q11.221 region is 2, refers to the fragment size of 15.35 Mb	#
2	UCB	46，X,+mar[22]/45,X[20]	52.4%/47.6%	A normal X chromosome. with additional material attached at Xq13.2	62 Mb loss of Xp22.33q11.1, 68 Mb loss of Xq21.31q28	The result shows 45,X, and with a duplication in Xq11.1q21.31 refers to the fragment size of 25.8 Mb	Healthy
3	UCB	46,X,+mar[21]/46,XY[24]	46.7%/53.3%	X, idic(Y) (p11.2)/XY mosaicism	The copy number of Ypter-q11.221 region is 2, refers to the fragment size of 16 Mb. The copy number of Yq11.221q11.23 region is 0, refers to the fragment size of 12.6 Mb	The copy number of Yq11.221q11.23 region is 0.48, refers to the fragment size of 11.9 Mb. The copy number of Yp11.31p11.2 region is 2, refers to the fragment size of 4.7 Mb. The copy number of Yp11.2 region is 1.67, refers to the fragment size of 1.4 Mb. The copy number of Yq11.21q11.221 region is 1.54, refers to the fragment size of 2.1 Mb	TOP
4	UCB	46,X,+mar[47]/45,X[3]	94%/6%	idic (Y) (p11.2) /X mosaicism	The copy number of Ypter-q11.221 region is 2, refers to the fragment size of 18.1 Mb. The copy number of Yq11.221q11.23 region is 0, refers to the fragment size of 10.3 Mb	The copy number of Yq11.221q11.23 region is 0, refers to the fragment size of 9.5 Mb. The copy number of Yp11.31q11.221 region is 2, refers to the fragment size of 15.6 Mb	TOP
5	UCB	45,X[24]/46,XX[81]	22.9%/77.1%	X/XX mosaicism, the ratio of XX is higher than X	45,X /46,XX, the ratio of mosaicism is about 30%	It suggested of a female fetus that was mosaicism, and the copy number of X chromosome was reduced, with a copy number of 1.61, involving the entire X chromosome	TOP
6	UCB	47,XXX[51]/45,X[49]	51%/49%	X/XX mosaicism	It suggested of a female fetus that was mosaicism, and the copy number of X chromosome was reduced, with a copy number of 1.7, involving the entire X chromosome	It suggested of a female fetus that was mosaicism, and the copy number of X chromosome was reduced, with a copy number of 1.71, involving the entire X chromosome	#
7	UCB	46,X,add(X)(p22)[69]/45,X[31]	69%/31%	A normal X chromosome, with additional material attached at Xp22 /X mosaicism	The copy number of Xp22.33p21.3 region is 1.87, refers to the fragment size of 25.8 Mb. The copy number of Xp21.3q28 region is 1.4, refers to the fragment size of 129.2 Mb	It suggested of a female fetus that was mosaicism, and the copy number of Xp21.3q28 region was 1.71, refers to the fragment size of 129.05 Mb	#
8	UCB	45,X[12]/47,XXX[8]/46,XX[60]	15%/10%/75%	XX	45,X /46,XX, the ratio of mosaicism is less than 30%	It suggested of a female fetus that was mosaicism, and the copy number of X chromosome was reduced, with a copy number of 1.92, involving the entire X chromosome	#
9	UCB	46,X,psuidic(X)(q28)[45]/45,X[25]	64.3%/35.7%	X	–	45,X	Healthy
10	UCB	47, XXY[6]/46,XY[34]	15%/85%	XY	–	The results showed that the ratio of X:Y was 1.15:1	TOP
11	AF	46,X,+mar[59]/45,X[6]	90.8%/9.2%	X, idic (Y) (p11.2) /X mosaicism	The copy number of pseudoautosomal region in X/Y is 3. The copy number of Yp11.31q11.21 region is 2, refers to the fragment size of 15.8 Mb. The copy number of Yq11.21q11.23 region is 0, refers to the fragment size of 10.3 Mb	The copy number of Yp11.31q11.221 region is 2, refers to the fragment size of 15.6 Mb. The copy number of Yq11.221q11.223 region is 0, refers to the fragment size of 6.25 Mb	#
12	AF	46,X,+mar[21]/45,X[12]	63.6%/36.4%	A normal X chromosome,with additional material attached at Xq13.2/X mosaicism	56 Mb loss of Xp22.33q11.21, 75 Mb loss of Xq21.1q28. The copy number of Xp11.2q21.1 region is 1.65, refers to the fragment size of 23 Mb	45,X, and the copy number of Xp11.21q21.1 region is 1.64, refers to the fragment size of 23.2 Mb	#
13	AF	46,X,+mar[9]/45,X[62]	12.7%/87.3%	X, idic (Y) (p11.2q11.222) /X mosaicism	The copy number of Y q11.221q11.222 region is 2, refers to the fragment size of 2.8 Mb. The copy number of Y q11.222q11.23 region is 0, refers to the fragment size of 7.7 Mb. The copy number of Yp11.31q11.221 region is 0.75	The copy number of Yp11.31p11.2 region is 0, refers to the fragment size of 4.65 Mb. The copy number of Yp11.2 region is 0.86, refers to the fragment size of 1.4 Mb. The copy number of Yq11.21q11.221 region is 0.75, refers to the fragment size of 2.5 Mb. The copy number of Yq11.222q11.23 region is 0, refers to the fragment size of 7.4 Mb. The copy number of Yq11.221 region is 1.44, refers to the fragment size of 2.4 Mb	#
14	AF	46,X,+mar[22]/45,X[40]	35.5%/64.5%	X	45,X	45,X	#
15	AF	45,X[43]/46,XX[19]	69.4%/30.6%	X/XX mosaicism	It suggested of a female fetus that was mosaicism, with a copy number of 1, which the rate of mosaicism is about 70%	It suggested of a female fetus that was mosaicism, and the copy number of X chromosome was reduced, with a copy number of 1.33, involving the entire X chromosome	#
16	AF	45,X[4]/46,XX[79]	4.8%/95.2%	XX	It suggested of a female fetus that was mosaicism, with the rate of mosaicism about 24%	It suggested of a female fetus that was mosaicism, and the copy number of X chromosome was reduced, with a copy number of 1.83, involving the entire X chromosome	Healthy
17	AF	45,X[34]/47,XXX[11]	75.6%/24.4%	X/XX mosaicism	It suggested of a female fetus that was mosaicism, and the copy number of X chromosome was reduced, with a copy number of 1.5, involving the entire X chromosome	It suggested of a female fetus that was mosaicism, and the copy number of X chromosome was reduced, with a copy number of 1.6, involving the entire X chromosome	TOP
18	AF	46,X,psuidic(Y)(p11.2)[63]/45,X[37]	63%/37%	X/XY mosaicism	The copy number of Xp22.33 or Yp11.32p11.31 region is 1–2. The copy number of Yp11.31q11.23 region is 0–1	The copy number of X chromosome was reduced, with a copy number of 1, involving the entire X chromosome. The ratio of X:Y was 1.15:1	#
19	AF	47,XXX[62]/45,X[18]	77.5%/22.5%	XX	It suggested of a female fetus that was mosaicism, and the copy number of X chromosome was increased, with a copy number of 2.3, involving the entire X chromosome	It suggested of a female fetus that was mosaicism, and the copy number of X chromosome was reduced, with a copy number of 2.25, involving the entire X chromosome	TOP
20	AF	45,X[31]/47,XYY[28]/46,XY[22]	38.3%/34.5%/27.2%	a normal X chromosome. with additional material attached at Xp22	It suggested of a male fetus that was mosaicism, and the copy number of Y chromosome was increased, with a copy number of 1.3	–	TOP
21	AF	45,X[25]/46,XX[65]	27.8%/72.2%	X/XX mosaicism	It suggested of a female fetus that was mosaicism, and the copy number of X chromosome was reduced, with a copy number of 1.7, involving the entire X chromosome. In addition, the copy number on the 16p12.2 chromosome is 1, refers to the fragment size of 702 kb	–	TOP
22	AF	45,X[20]/46,XX[56]	26.3%/73.7%	X/XX mosaicism	It suggested of a female fetus that was mosaicism, which the rate of mosaicism is about 20%. The copy number of X chromosome was reduced, with a copy number of 1.8	It suggested of a female fetus that was mosaicism, and the copy number of X chromosome was reduced, with a copy number of 1.79, involving the entire X chromosome	#
23	AF	45,X[8]/46,XX[76]	9.5%/90.5%	X/XX mosaicism	It suggested of a female fetus that was mosaicism, which the rate of mosaicism is 50% and involves the whole X chromosome	It suggested of a female fetus that was mosaicism, and the copy number of X chromosome was reduced, with a copy number of 1.7, involving the entire X chromosome	#
24	AF	45,X[28]/46,X，del(X)(q22)[24]	53.8%/46.2%	X/X,del(X)(q27) mosaicism	–	It suggested of a female fetus that was mosaicism, and the copy number of Xp21.3q28 region is 1.19 refers to the fragment size of 71.45 Mb	#
25	AF	45,X[7]/46,XX[99]	6.6%/93.4%	XX	–	46,XX	#
26	AF	45,X[9]/46,XY[105]	7.9%/92.1%	X/X,del(Y)(q11)	–	The results showed that the ratio of X:Y was 1:0.85	Healthy
27	AF	45,X[29]/46,XX[46]	38.7%/61.3%	XX	–	It suggested of a female fetus that was mosaicism, and the copy number of X chromosome was reduced, with a copy number of 1.55, involving the entire X chromosome	#
28	AF	47,XYY[26]/46,XY[56]	31%/69%	X,add(Y)(p11)/XY mosaicism	–	The results showed that the ratio of X:Y was 0.5:0.615. The copy number of Yp11.32q12 region is 1.23, refers to the fragment size of 59.35 Mb	#
29	AF	45,X[7]/46,XX[73]	8.8%/91.2%	XX	–	–	#
30	AF	45,X[10]/46,XX[23]	30.3%/69.7%	X/XX mosaicism	–	It suggested of a female fetus that was mosaicism, and the copy number of X chromosome was reduced, with a copy number of 1.74, involving the entire X chromosome	#
31	AF	47,XXY[12]/46,XY[36]	25%/75%	XY	–	The results showed that the ratio of X:Y was 0.52: 0.5. The copy number of Xp22.33q28 region was 1.04, refers to the fragment size of 155.2 Mb	healthy

*AF* amniotic fluid, *UCB* umbilical cord blood, *TOP* termination of pregnancy

^#^loss to follow-up, –undetected

At the same time, sex chromosome abnormalities were detected in a total of 21 cases tested by SNP-array, with the lowest proportion of mosaicism being 4.8%. Among them, 15 cases were diagnosed as sex chromosome abnormalities with indications of mosaicism, and 6 cases were detected as sex chromosome abnormalities without indications of mosaicism (cases 1, 2, 3, 4, 11, 14) (Table 2).

CNV-seq was performed on 28 cases, and sex chromosome abnormalities was detected in all of them except one sample (case 25), with the lowest proportion of mosaicism being 4.8%. Among them, there were 21 cases with sex chromosome abnormalities indicated of mosaicism, and sex chromosome abnormalities was detected but without indications of mosaicism in 6 cases (case 1, 2, 4, 9, 11, 14).

### Sex chromosome abnormality detected by karyotyping and BoBs assay

Sex chromosome mosaicism were diagnosed in all 31 samples by karyotype analysis, and the proportion of mosaicism range from 4.8% to 94%. Among the 31 cases, 11 cases were detected by prenatal BoBs, while 8 cases (cases 8, 10, 16, 19, 25, 27, 29, and 31) were not detected. The proportion of abnormal cells with mosaicism was 4.8–38.7%. The prenatal BoBs assay was able to detect structurally abnormal mosaicism in all 4 diagnosed cases, as well as mosaicism of sSMC in all 8 diagnosed cases.

### Sex chromosome abnormality detected by SNP-array and BoBs assay

The results of prenatal BoBs were consistent with the results of the SNP-array in 13 cases, with sex chromosome abnormalities detected in all cases. In 4 cases (cases 1, 3, 5, and 11), the results of prenatal BoBs indicated mosaicism, while the results of SNP-array identified the specific location of the abnormal occurrence without suggesting mosaicism. 3 cases (cases 8, 16, and 19) showed no obvious abnormalities in the results of prenatal BoBs, while SNP-array results suggested mosaicism. Therefore, prenatal BoBs have comparable sensitivity as SNP-array in detecting structural abnormal mosaicism and chromosomal abnormal markers.

### Sex chromosome abnormality detected by CNV-seq and BoBs assay

**Fig. 2:**
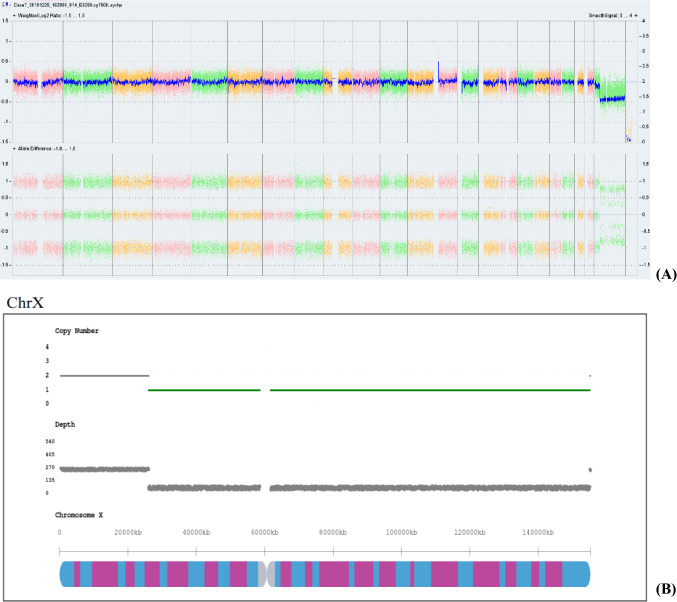
Sex chromosomal abnormalities detected in case7 by single nucleotide polymorphism microarray (SNP-array) and copy number variation sequencing (CNV-Seq). **A** Results of SNP-array showed the copy number of Xp22.33p21.3 region is 1.87, refers to the fragment size of 25.8 Mb. The copy number of Xp21.3q28 region was 1.4, refers to the fragment size of 129.2 Mb. **B** Results of CNV-Seq suggested of a female fetus that was mosaicism, and the copy number of Xp21.3q28 region was 1.71, refers to the fragment size of 129.05 Mb

**Fig. 3:**
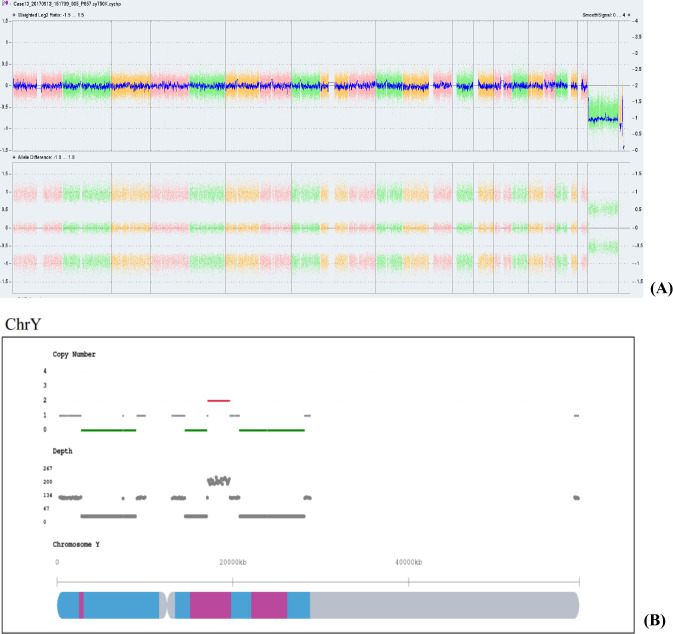
Sex chromosomal abnormalities detected in case13 by single nucleotide polymorphism microarray (SNP-array) and copy number variation sequencing (CNV-Seq). **A** Results of SNP-array showed the copy number of Y q11.221q11.222 region is 2, refers to the fragment size of 2.8 Mb. The copy number of Y q11.222q11.23 region is 0, refers to the fragment size of 7.7 Mb. The copy number of Yp11.31q11.221 region is 0.75. **B** Results of SNP-array showed the copy number of Yp11.31p11.2 region is 0, refers to the fragment size of 4.65 Mb. The copy number of Yp11.2 region is 0.86, refers to the fragment size of 1.4 Mb. The copy number of Yq11.21q11.221 region is 0.75, refers to the fragment size of 2.5 Mb. The copy number of Yq11.222q11.23 region is 0, refers to the fragment size of 7.4 Mb. The copy number of Yq11.221 region is 1.44, refers to the fragment size of 2.4 Mb

The results of prenatal BoBs were consistent with the results of the CNV-seq in 18 cases, with sex chromosome abnormalities detected in all cases except one sample (case 25), and two cases (case 9, 14) were detected without indications of mosaicism. In 3 cases (case 1,4, 11), the result of prenatal BoBs suggested sex chromosome mosaicism, but the location of the abnormal occurrence could not be identified, while the results of CNV-seq identified the specific abnormal segment without suggesting mosaicism, which was consistent with the results of SNP-array. 6 cases (cases 8, 10, 16, 19, 27and 31) showed no obvious abnormalities in the results of prenatal BoBs, while the results of CNV-seq suggested mosaicism with different proportions, which were all consistent with the results of SNP-array except for 3 cases that were not performed by SNP-array. SNP-array and CNV-seq were performed on 19 samples at the same time, and the results were inconsistent in 2 cases (Case 7, 13). In case7, the segment of Xp22.33p21.3 indicated mosaicism by SNP-array, while the results of CNV-seq were not (Fig. 2). In case 13, the result of abnormal fragment of CNV-seq was slightly different from that of SNP-array, which considering that read distribution was different (Fig. 3).

### Pregnancy outcomes

Clinical evaluation of pregnancy outcomes was reported in 13 of 31 cases (42%) with sex chromosome mosaicism. Normal outcomes were reported in 5 cases (16%), while termination of pregnancy occurred in 8 cases.

## Discussion

The prenatal BoBs assay was recently introduced for the sake of detecting abnormalities of chromosomes 13, 18, 21, X, and Y, as well as nine specific significant microdeletion syndromes. Several studies have been published on its performance in detecting aneuploidies and microdeletions (Gross et al. 2011; Huang et al. 2017; Li et al. 2018; Rosenfeld et al. 2014). According to retrospective analysis, the global mosaic detection rate is 20% or greater using prenatal BoBs assay (Cheng et al. 2013), but there is no research on the detection of sex chromosome mosaicism by prenatal BoBs.

Our study is the first study to date to evaluate the potential use of prenatal BoBs for prenatal diagnosis of sex chromosome mosaicism. Prenatal BoBs diagnosed 23 cases of sex chromosome abnormality, and demonstrated a detection limit of 6% in those same samples. 19 cases were tested for sex chromosome abnormality and had results that indicated mosaicism. 4 cases had results that suggested sex chromosome abnormality but did not indicate mosaicism. Among those cases with abnormal mosaicism, 11 cases were detected by prenatal BoBs. All cases of structurally abnormal sex chromosome mosaicism were detected by prenatal BoBs, and sSMC could further identify the origin of abnormal chromosomes within the range of detection. Therefore, the prenatal BoBs technique has a high detection rate for sex chromosome mosaicism.

Twenty-one samples were also tested by SNP-array. Sex chromosome abnormalities were detected in all those samples, with a threshold proportion of mosaicism of 4.8%. Among them, 15 cases were diagnosed as sex chromosome abnormalities and indicated mosaicism, while 6 cases were detected as sex chromosome abnormalities but did not indicate mosaicism. CNV-seq was performed on 28 cases, and sex chromosome abnormalities were detected in all of them except case25, with the lowest proportion of mosaicism being 4.8%. There were 21 cases with sex chromosome abnormalities indicative of mosaicism, while 6 cases were detected as sex chromosome abnormalities without indications of mosaicism. The design region probe of prenatal BoBs is different from SNP-array and CNV-seq that covering the whole genome, in which the sex chromosome signals are generated by direct calculation of fluorescence values by prenatal BoBs, resulting in a slightly more obvious signal trend that may be the reason for the detection rate of prenatal BoBs. The scope of prenatal BoBs is limited by the location and number of probes, so its results can only infer the presence of mosaicism, whereas SNP-array and CNV-seq can specify the proportion and composition of that mosaicism. As a molecular detection technology, prenatal BoBs can only detect dose changes in its probe area. The ratio of specimen fluorescence to reference fluorescence is not calculated in accordance with real cell cloning, so it is limited in its ability to detect low proportions of mosaicism. Therefore, mosaicism cannot be accurately detected. Furthermore, results may vary depending on sample materials and fluctuations of sex chromosome probes, which makes the ratio of types of mosaicism fluctuate successively, rendering it impossible to distinguish between multiple mosaicism types (e.g., quantitatively balanced 45,X/47,XYY).

There were some differences in detection rates and situations of detection among the four detection methods. Prenatal BoBs, SNP-array and CNV-seq directly extracted amniotic fluid or cord blood DNA for testing and karyotyping was analyzed by cell culture, sample source was different. The difference in the proportion of mosaicism may be due to the following reasons: (1) During the culture process, the proportion of cell lines was not consistent with that before culture. (2) There may be a small number of maternal cells in amniotic fluid cells, cells of maternal and fetal were grown at the same time, which affects the mosaicism proportion. (3) Aberration occurred during the in cells culture in vitro. The amniotic fluid samples used for karyotyping, prenatal BoBs, SNP-array and CNV-seq testing are not in the same tube, just like a sampling survey. Amniotic fluid of mosaicism in the germ layer is randomness and variable.

Mosaicism is identified in about 50% of females with Turner syndrome, and an estimated subset of 6–12% of all Turner syndrome patients will be mosaic with Y-chromosomal elements (Armstrong et al. 2020). Genetic counselling and clinical management of sex chromosome mosaicism in prenatal diagnosis remain challenging due to variable phenotype presentation and unclear significance of symptoms. At present, none of the sex chromosome mosaicism cases we followed up with post-study showed obvious growth and development abnormalities. However, because the symptoms of sex chromosome mosaicism are likely be clinically evident only after the onset of puberty, it is necessary to continue with follow-ups all the way to adulthood (Artemis, et al. 2018; Calanchini et al. 2020; Gravholt et al. 2017; Lim et al. 2017; Prakash et al. 2018). Mosaicism remains a challenge to address in genetic counseling, which means that greater caution should be used in prenatal counseling. The use of more comprehensive molecular diagnostic assays in combination of karyotyping to detect sex chromosome mosaicism is highly recommended, to determine the type and proportion of mosaicism more accurately. The purpose of this recommendation is to provide a more reliable information base on which clinicians can guide genetic counseling.

This study evaluated the effectiveness of prenatal BoBs for detecting sex chromosome mosaicism in prenatal diagnosis, benchmarking against karyotyping, SNP-array and CNV-seq as a reference. Of the 31 positive mosaic cases, the levels of sex chromosome mosaicism detected by prenatal BoBs ranged from 6 to 94% compared to the ratio from 4.8% to 94% by SNP-array, CNV-seq and karyotyping. Karyotyping has been used as the golden standard to detect chromosomal abnormalities in prenatal diagnosis with a maximum resolution of 3 Mb (Vermeesch et al. 2007). In general, the low limit of true mosaicism detected by karyotyping is about 5% (Hook 1977). In this study, the proportion of mosaicism in karyotyping was as low as 4.8%, which was similar to that reported in the literature. However, the technique requires up to 2 weeks from amniocentesis to diagnosis and high requirements on the professional level of technicians; moreover, it is susceptive to contamination in the cell culture process. Therefore, new techniques are urgently needed in clinical to supplement the limitations of karyotyping. With the development of molecular biology, a variety of molecular diagnostic techniques have emerged to be used in clinical. In recent years, SNP-array and CNV-seq had been widely applied to detect chromosome variation in the whole genome, and the detectable levels were 30% to 70% by Affymetrix arrays (Pinto et al. 2018; Zahir and Marra, 2015), and the levels of mosaicism detected by CNV-seq ranged from 6 to 92% (Ma et al. 2021). In our study, the proportion of sex chromosome mosaicism detected by SNP-array and CNV-seq were 4.8%, especially for sSMC, SNP-array and CNV-seq can clarify its source and segment size, they supplemented the limited resolution of conventional karyotyping.

In conclusion, the prenatal BoBs assay is a rapid and accurate molecular technology, and its method of diagnosis using uncultured amniotic fluid can be a good complement to karyotyping, with the detection level of sex chromosome mosaicism reaching as low as 6%, and suggested the prenatal BoBs has comparable sensitivity as SNP-array in detecting structural abnormal mosaicism and chromosomal abnormal markers. Because of the limited numbers of marker sites, the prenatal BoBs assay can indicate the site of sex chromosome abnormality, but the specific size of the abnormality fragment cannot be determined. Thus, the prenatal BoBs assay alone is not sufficient for prenatal diagnosis, it is necessary to combine cellular and other molecular genetic methods such as SNP-array and CNV-seq for a more accurate diagnosis of sex chromosome mosaicism.
